# The Use of Patient-Derived Organoids in the Study of Molecular Metabolic Adaptation in Breast Cancer

**DOI:** 10.3390/ijms251910503

**Published:** 2024-09-29

**Authors:** Natalija Glibetic, Scott Bowman, Tia Skaggs, Michael Weichhaus

**Affiliations:** 1 Laboratory of Molecular Cancer Research, School of Natural Sciences and Mathematics, Chaminade University of Honolulu, Honolulu, HI 96816, USA; natalija.glibetic@chaminade.edu (N.G.); scott.bowman@student.chaminade.edu (S.B.); tia.skaggs@student.chaminade.edu (T.S.); 2 The IDeA Networks of Biomedical Research Excellence (INBRE) Program, School of Natural Sciences and Mathematics, Chaminade University, Honolulu, HI 96816, USA; 3 United Nations CIFAL Honolulu Center, Chaminade University, Honolulu, HI 96816, USA; 4 Undergraduate Program in Biochemistry, School of Natural Sciences and Mathematics, Chaminade University, Honolulu, HI 96816, USA; 5 Undergraduate Program in Biology, School of Natural Sciences and Mathematics, Chaminade University, Honolulu, HI 96816, USA

**Keywords:** breast cancer, organoid, metabolism, glycolysis, lipid metabolism, optical metabolic imaging, MALDI-MSI, tumor microenvironment

## Abstract

Around 13% of women will likely develop breast cancer during their lifetime. Advances in cancer metabolism research have identified a range of metabolic reprogramming events, such as altered glucose and amino acid uptake, increased reliance on glycolysis, and interactions with the tumor microenvironment (TME), all of which present new opportunities for targeted therapies. However, studying these metabolic networks is challenging in traditional 2D cell cultures, which often fail to replicate the three-dimensional architecture and dynamic interactions of real tumors. To address this, organoid models have emerged as powerful tools. Tumor organoids are 3D cultures, often derived from patient tissue, that more accurately mimic the structural and functional properties of actual tumor tissues in vivo, offering a more realistic model for investigating cancer metabolism. This review explores the unique metabolic adaptations of breast cancer and discusses how organoid models can provide deeper insights into these processes. We evaluate the most advanced tools for studying cancer metabolism in three-dimensional culture models, including optical metabolic imaging (OMI), matrix-assisted laser desorption/ionization mass spectrometry imaging (MALDI-MSI), and recent advances in conventional techniques applied to 3D cultures. Finally, we explore the progress made in identifying and targeting potential therapeutic targets in breast cancer metabolism.

## 1. Introduction

In 2022, 42,211 women in the U.S. died from breast cancer; it continues to be one of the most prevalent forms of diagnosed cancers in women, with a one in eight (13%) lifetime risk of developing the disease [1,2]. Metabolic alterations in tumors were one of the first discoveries in cancer biology and can be categorized into six hallmarks: (1) deregulated glucose and amino acid uptake, (2) utilization of opportunistic nutrient acquisition methods, (3) reliance on glycolysis/tricarboxylic acid (TCA) cycle intermediates for biosynthesis and nicotinamide adenine dinucleotide phosphate (NADPH) production, (4) heightened nitrogen demand, (5) alterations in gene regulation driven by metabolites, and (6) metabolic interactions with the microenvironment [3,4,5,6]. Since cancer cells frequently depend on reprogramming metabolic pathways to support their rapid proliferation and survival, there is interest in targeting cancer metabolism as a therapeutic strategy. However, traditional cancer research methods, while invaluable, often fall short in replicating the complex TME observed in patients where these metabolic dependencies occur. One promising approach to addressing these challenges is the use of patient-derived tumor organoids (PDTO).

PDTOs are self-organized tissue or stem cell-derived three-dimensional (3D) cultures that have the ability for self-renewal. In contrast to two-dimensional (2D) cell cultures, which typically contain one cell type on a flat surface, organoids better recapitulate tissues through more accurate structural and functional characteristics [7,8]. To date, a wide variety of organoid models have been developed, including brain [7,9,10,11,12], intestine [13,14,15,16], liver [17,18,19], kidney [20,21,22,23], pancreas [24,25,26], prostate [25,26,27,28], and tumor [29,30,31,32,33]. Culturing in 3D conditions allows organoids to mimic original tissue structures, making them broadly useful for a variety of disease studies. PDTOs derive from various sources, such as tumor biopsies and circulating tumor cells, and have been instrumental in exploring metabolic alterations in cancers, including breast cancer [31]. Recent studies have expanded the use of PDTOs to investigate breast cancer metabolism. Furthermore, advanced approaches like PDTO-immune co-culture models have emerged to more accurately represent the in vivo TME. These models have the potential to enhance understanding of tumor metabolism and immune interactions. Despite this potential, adopting PDTOs for metabolic investigation has not been widespread.

In this review, we examine the potential applications and limitations of PDTO cultures for studying metabolic diseases in cancer, with a focus on breast cancer. PDTOs provide a complex alternative to traditional two-dimensional cultures by more accurately reflecting the TME, tumor heterogeneity, and metabolic dysregulations typically found in tumor tissue. First, we provide an excerpt of the current research into breast cancer metabolism, which may be suitable for investigation in PDTOs. Next, we assess advanced techniques for analyzing breast cancer metabolism in PDTOs, such as OMI, MALDI-MSI, and multi-omic approaches. Finally, we discuss recent advancements in identifying and targeting therapeutic opportunities in breast cancer metabolism.

## 2. Metabolic Dysregulation in Cancer

**Figure 1:**
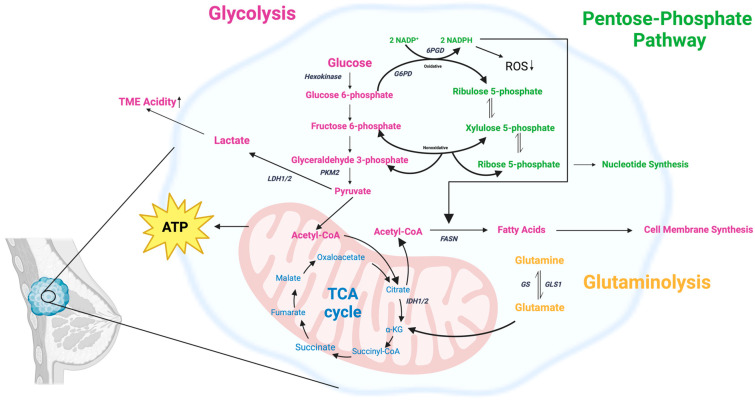
A non-exhaustive representation of key metabolic pathway alterations in breast cancer cells, including glycolysis, the TCA cycle, the pentose phosphate pathway (PPP), and glutaminolysis. Breast cancer cells show increased glycolysis, converting glucose to lactate (Warburg effect), contributing to an acidic TME. Key enzymes such as hexokinase (HK), pyruvate kinase M2 (PKM2), and lactate dehydrogenase (LDH1/2) are involved. The oxidative PPP is upregulated, increasing ribose-5-phosphate and NADPH production. Alterations in TCA cycle enzymes (IDH1/2) and glutamine metabolism (GLS1) support biosynthesis and proliferation. Fatty acid synthesis is upregulated, with fatty acid synthase (FASN) producing fatty acids from acetyl-CoA. Created with BioRender.com.

One of the foundational discoveries in cancer biology is the identification of metabolic alterations in tumors, which has profoundly shaped our understanding of cancer progression and treatment [4,5]. Among these alterations, the most striking is the dysregulated carbohydrate metabolism characterized by increased glycolytic activity, even in the presence of sufficient oxygen—a process commonly referred to as the Warburg effect or aerobic glycolysis [34,35,36]. This phenomenon, where cancer cells preferentially utilize glycolysis over oxidative phosphorylation (OXPHOS) to generate energy, is a near-universal feature of cancers, including breast cancer [37,38]. Unlike normal cells, which rely primarily on OXPHOS under aerobic conditions to maximize ATP production, cancer cells exploit aerobic glycolysis to support their rapid growth and proliferation, providing both energy and metabolic intermediates needed for biosynthesis [39]. In addition to changes in glycolysis, various other metabolic alterations in breast cancer cells have been identified and will be introduced in the following sections (Figure 1).

### 2.1. Dysregulation in Glycolysis

**Figure 2:**
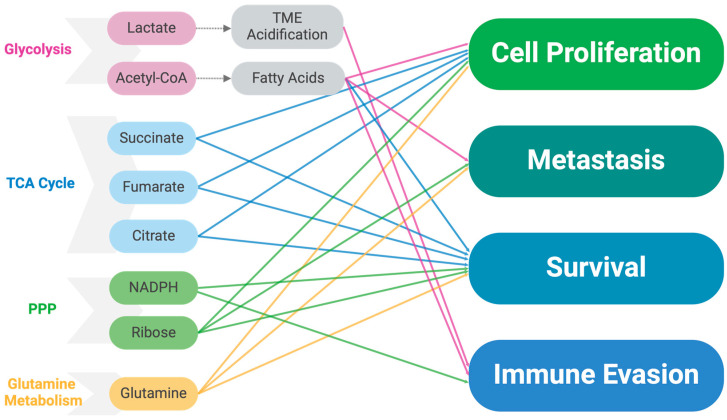
Metabolite influence on cancer cell behavior. Key metabolites from glycolysis, TCA cycle, PPP, and glutamine metabolism and their roles in driving cancer cell processes. Metabolites from these pathways contribute to cell proliferation, metastasis, survival, and immune evasion. Lactate and acetyl-CoA (glycolysis) promote TME acidification and fatty acid synthesis. Succinate, fumarate, and citrate (TCA cycle) support growth and survival. NADPH and ribose (PPP) are involved in biosynthesis and immune evasion, while glutamine fuels multiple processes. Colored arrows indicate specific metabolite influences on cellular behaviors. Created with BioRender.com.

Aerobic glycolysis, also known as the Warburg effect, is a metabolic adaptation seen in many cancer cells where glucose is preferentially converted to lactate even in the presence of sufficient oxygen. This metabolic shift results in a significantly increased intake of glucose by cancer cells compared to normal cells. There are several reasons for this change: first, cancer cells have higher metabolic rates than normal cells due to their rapid growth and proliferation demands, which require more energy and nutrients [40]. Second, while the conversion of glucose to lactate (aerobic glycolysis) is less efficient in ATP production than OXPHOS, it provides several advantages for cancer cell survival and growth. By relying on aerobic glycolysis, cancer cells can thrive in fluctuating oxygen conditions, such as those found in the hypoxic regions of solid tumors. This flexibility allows them to maintain energy production even when oxygen levels are low, which is crucial for survival in the dynamic TME [41,42]. Furthermore, aerobic glycolysis supports rapid cell growth and proliferation by providing a continuous supply of glycolytic intermediates. These intermediates are not only used for energy but are also diverted into various biosynthetic pathways essential for building cellular components necessary for cell division, such as nucleotides, amino acids, and lipids [43,44,45]. We will discuss the specific involvement of the pentose phosphate pathway (PPP) in Section 2.2. Another consequence of aerobic glycolysis is the excessive accumulation of lactate in the TME. This lactate buildup leads to acidosis, which can inhibit the immune response against the tumor, providing cancer cells with a form of immune evasion (Figure 2). Additionally, the acidic environment promotes tissue invasion and metastasis by enhancing the degradation of the extracellular matrix (ECM) and facilitating cancer cell migration to distant sites. [46]. We will discuss the effects on the TME specifically in Section 2.9.

Our understanding of how cancer cells accomplish sustained aerobic glycolysis is still forthcoming; however, some molecular mechanisms have been discovered. Hexokinase, particularly Hexokinase 2 (HK2), plays a crucial role in aerobic glycolysis in cancer cells by being the initial rate-limiting enzyme in the glycolytic pathway, catalyzing the phosphorylation of glucose to glucose-6-phosphate. This step is essential for glucose to be retained within the cell and used for glycolysis [47]. HK2-specific overexpression is routinely observed in breast tumor tissue compared to healthy breast tissue [48]. Modulating HK2 activity alters a cancer cell’s glycolytic rate, progression, and paclitaxel resistance in breast cancer cells [49,50,51]. In addition to metabolic effects, the inhibition of HK2 can also induce cell death mechanisms like pyroptosis and amplify immunogenic cell death [52].

In addition to HK2, the hypoxia-inducible form of 6-phosphofructo-2-kinase (PFKFB3) is another glycolytic enzyme implicated in promoting aerobic glycolysis in breast cancer [53]. The inhibition of PFKFB3 suppresses glucose metabolism and the growth of HER2+ breast cancer [54]. Another key glycolytic enzyme associated with aerobic glycolysis in cancer cells is pyruvate kinase M2 (PKM2). PKM2 is essential for promoting aerobic glycolysis by catalyzing the final step of the glycolytic pathway, converting phosphoenolpyruvate to pyruvate. Unlike its counterpart, PKM1, PKM2 can exist in a less active dimeric form, which slows down this final step of glycolysis, leading to the accumulation of upstream glycolytic intermediates. [55,56,57]. It is of particular interest that PKM2 can interact with various oncogene products, providing a direct pathway between genetic tumorigenesis and altered metabolic pathways in cancer cells. For example, CD44, a cell surface marker for cancer stem cells, interacts with PKM2 and thereby enhances the glycolytic phenotype of cancer cells [58]. PKM2 has also been implicated in mediating the cancer phenotype by a secondary function in transcription activation. For example, PKM2 can translocate to the nucleus, where it influences gene expression by acting as a coactivator for hypoxia-inducible factor 1-alpha (HIF-1α) and other transcription factors [59]. This nuclear function of PKM2 supports the transcription of genes involved in glycolysis and cell cycle progression, further promoting cancer cell survival and proliferation. We will discuss the influence of HIF-1α on oncogenic metabolism in more detail in Section 2.7.

An emerging modulator for aerobic glycolysis is pyruvate dehydrogenase kinase 1 (PDHK1), which phosphorylates and inactivates mitochondrial pyruvate dehydrogenase (PDH) and consequently pyruvate dehydrogenase complex (PDC). This effectively reduces the amount of pyruvate that may enter the mitochondria and be respirated off during OXPHOS. By limiting the entry of pyruvate into the TCA cycle, PDK supports the continuation of high glycolytic rates [60]. PDHK1 is upregulated by oncogenic signals such as M and HIF-1α [61]. PDK has become an attractive target for pharmaceutical intervention as its inhibition can lead to a shift back towards oxidative metabolism, reducing tumor growth and enhancing the effectiveness of cancer therapies [62].

### 2.2. Pentose Phosphate Pathway (PPP)

One of the important biosynthetic pathways that feed off the accumulated glycolytic intermediates is the PPP. The PPP diverts glucose flux to its oxidative branch, producing ribose-5-phosphate for nucleotide synthesis and reduced NADPH for reductive biosynthesis, such as fatty acid synthesis and detoxification of reactive oxygen species (ROS) by regenerating glutathione into its reduced form [63]. Fluctuating NADPH levels are one of the potential investigative targets we will discuss in Section 4.1. The oxidative branch of the PPP uses glucose-6-phosphate as its substrate. Upregulation of glucose-6-phosphate dehydrogenase (G6PD) is commonly observed in cancer cells, enhancing their ability to manage oxidative stress and sustain high rates of proliferation [64]. In breast cancer cells, the PPP is often upregulated to meet the demands for nucleotide and fatty acid synthesis, as well as to combat oxidative stress. This metabolic reprogramming supports rapid cell growth and survival [65]. The inhibition of key PPP enzymes, such as G6PD and 6-phosphogluconate dehydrogenase (6PGD), in breast cancer cells has been shown to reduce cell proliferation, induce oxidative stress, and decrease survival [66]. Elevated levels of ribose-5-phosphate and other PPP intermediates have been directly linked to enhanced DNA synthesis and cell cycle progression, highlighting the importance of ribose-5-phosphate in supporting rapid tumor growth (Figure 2) [67].

Increased NADPH production provides several advantages. The NADPH produced through the PPP helps maintain redox homeostasis by reducing ROS levels, thereby protecting cancer cells from oxidative damage and supporting their survival in hostile environments [68]. Enhanced NADPH production also supports fatty acid synthesis, which is vital for membrane biosynthesis and rapid cell proliferation in cancer cells [69]. Aerobic glycolysis provides ample precursor molecules for the PPP, while the adaptations in the pathway itself provide critical support for biosynthetic processes and antioxidant defenses, facilitating the aggressive growth and survival of cancer cells under various stress conditions.

### 2.3. Mitochondrial Changes and TCA Dysregulation

In addition to modulating glycolysis directly, changes in related metabolic pathways have been found to contribute to aerobic glycolysis while simultaneously providing further growth and progression advantages to cancer cells. Mitochondrial DNA (mtDNA) is more susceptible to mutations due to limited repair mechanisms compared to nuclear DNA [70]. Mutations in mtDNA polymerase γ (POLG) lead to decreased OXPHOS, mtDNA depletion, increased ROS, and enhanced invasiveness in breast cancer cells [71]. Similarly, mitochondrial DNA mutations reduce the efficiency of the mitochondrial respiratory chain, leading to impaired energy production and increased ROS generation, contributing to cancer progression [72]. These changes in mitochondrial dysfunction further contribute to enhancing glycolytic rates. Since mitochondrial dysfunction increases ROS production due to defective OXPHOS, this can cause further DNA damage and mutations, driving cancer progression and metastasis [73]. Mutations in mtDNA and the resulting metabolic adaptations, such as increased lactate production and reduced oxygen consumption, confer resistance to apoptosis and chemotherapy, contributing to tumor survival and growth [74].

In addition to mitochondrial DNA mutations, mitochondrial changes in the TCA cycle flux are commonly observed in cancer cells, including breast cancer, due to mutations in TCA cycle enzymes [75]. For example, genetic alterations in enzymes like succinate dehydrogenase (SDH), fumarate hydratase (FH), and isocitrate dehydrogenase (IDH) are linked to tumor development and progression [76,77,78]. One of the consequences of these adaptations is that the TCA slows its progression and accumulates intermediates, such as citrate and succinate, which can create a backlog into glycolysis, further enhancing aerobic glycolysis in cancer cells [79,80]. Additionally, like the advantages of accumulating glycolytic intermediates for biosynthesis, the accumulation of TCA intermediates supplies essential metabolic intermediates for anabolic processes, primarily amino acid synthesis, supporting rapid cell growth (Figure 2) [77]. The importance of TCA dysregulation in cancer cells may be further emphasized by its regulation within the wider framework of cancer development. For example, BRCA1, a tumor suppressor, reprograms breast cancer cell metabolism, enhancing the TCA cycle and OXPHOS while inhibiting glycolysis, countering the Warburg effect [81]. Additionally, a dysregulated TCA cycle is associated with resistance to therapies, such as endocrine treatments in ER+ breast cancer, where impaired SDH activity leads to succinate accumulation and therapy resistance [81].

### 2.4. Dysregulated Lipid Metabolism

In addition to aerobic glycolysis, various associated metabolic pathways are dysregulated in cancer cells. Cancer cells often exhibit deregulated lipid metabolism, characterized by increased lipogenesis. This process, particularly the upregulation of fatty acid synthase (FASN), plays a crucial role in supporting the rapid growth and survival of cancer cells, including those in breast cancer. The upregulation of FASN and other lipogenic enzymes is a common feature in cancer cells, supporting their rapid proliferation by providing necessary lipids for membrane synthesis and energy production (Figure 2). This upregulation is often driven by oncogenic signaling pathways such as the PI3K/Akt and MAPK pathways [82]. FASN provides the building blocks for cell membrane synthesis and energy storage, which are crucial for cancer cell proliferation and survival [83]. The inhibition of FASN in breast cancer stem cells has been shown to significantly reduce their self-renewal and growth capabilities, highlighting the enzyme’s essential role in maintaining the malignant phenotype of these cells [84]. HBXIP, an oncoprotein, has been shown to contribute to abnormal lipid metabolism in breast cancer by activating the LXRs/SREBP-1c/FAS signaling cascade, which enhances lipogenesis and promotes tumor growth [85]. The importance of dysregulated lipid metabolism goes beyond energy metabolism and biosynthesis of membranes. Inhibiting key enzymes involved in lipogenesis, such as FASN, acetyl-CoA carboxylase, and ATP-citrate lyase, has shown promise in limiting cancer cell proliferation and survival [86]. Lipogenesis inhibitors can induce apoptosis and cell cycle arrest in breast cancer cells, further emphasizing the potential of targeting lipid metabolism as a cancer treatment strategy [87].

While much research has focused on the synthesis of novel fatty acids in cancer cells, concerning catabolic energy metabolism, fatty acid oxidation (FAO) is similarly dysregulated to supply energy to fast-growing cancer cells. In breast cancer, particularly in triple-negative breast cancer (TNBC), the upregulation of FAO pathways has been observed. MYC-overexpressing TNBC cells rely heavily on FAO for their energy needs, and the inhibition of FAO significantly impairs tumor growth and survival [88]. The JAK/STAT3 signaling pathway is crucial for regulating FAO in breast cancer stem cells (BCSCs). Inhibiting this pathway reduces FAO activity, leading to decreased BCSC self-renewal and increased sensitivity to chemotherapy [89]. FAO provides a significant source of ATP for cancer cells, especially under metabolic stress or in the TME where glucose availability might be limited. This energy supply is critical for maintaining cellular functions and supporting rapid proliferation [90]. The products of FAO are used in membrane synthesis, which is essential for the expansion and integrity of rapidly dividing cells. In breast cancer, lipidomic studies have shown increased levels of specific fatty acids incorporated into membrane phospholipids, correlating with tumor progression and poor prognosis [83]. Given the reliance of certain breast cancer subtypes on FAO, targeting this metabolic pathway offers therapeutic potential. Pharmacological inhibition of FAO has been shown to reduce tumor growth and improve outcomes in preclinical models [88]. Combining FAO inhibitors with other treatments, such as chemotherapy, can enhance the overall therapeutic efficacy by simultaneously targeting multiple metabolic vulnerabilities in cancer cells [91].

### 2.5. Dysregulated Glutamine Metabolism

The TCA cycle relies on several intermediates derived from the carbon skeletons of amino acids. Of particular importance is α-ketoglutarate, which can be generated from glutamine, the most abundant amino acid in the bloodstream [92]. Thus, glutamine becomes an important energetic substrate for cancer metabolism. Cancer cells, including breast cancer cells, often exhibit increased glutaminolysis, converting glutamine to glutamate and subsequently to α-ketoglutarate, which fuels the TCA cycle (Figure 2). This process is crucial for providing energy and biosynthetic precursors for rapidly proliferating cells [93]. In breast cancer, particularly TNBC, glutaminase inhibition leads to significant tumor growth reduction [94,95]. Targeting this enzyme disrupts the metabolic flexibility of cancer cells, leading to reduced proliferation and increased sensitivity to other treatments [96,97]. Glutaminolysis also helps maintain redox balance by producing glutathione, a major cellular antioxidant, thus protecting cancer cells from oxidative stress and promoting survival [98]. Invasive breast cancer cells utilize glutaminolysis not only for growth but also to promote invasiveness. The conversion of glutamine to glutamate and its subsequent release activates metabotropic glutamate receptors, enhancing invasive behaviors like matrix metalloprotease recycling, which facilitates tissue invasion [99]. Glutamine metabolism has also been linked with resistance to various common therapies. Studies have identified the role of glutamine metabolism in breast cancer resistance to taxol [100], antiestrogen therapy, and tamoxifen [101]. In all cases, targeting glutamine metabolism, either through the inhibition of glutaminase 1 (GLS1) or the use of metabolic inhibitors, has been shown to resensitize resistant cells to treatment.

In addition to glutaminolysis, glutamine synthesis has likewise been associated with increased metabolic plasticity in breast cancer cells. Glutamine synthetase (GS) plays a crucial role in determining the glutamine dependence of breast epithelial cells, with luminal-type cells being more glutamine-independent due to the lineage-specific expression of GS [102]. This is further supported by the finding that the epithelial-to-mesenchymal transition (EMT) promotes glutamine independence by suppressing GLS2 expression, which encodes a glutaminase [103]. The regulation of GS by 1,25-dihydroxyvitamin D (1,25D) also influences glutamine metabolism in breast epithelial cells, with 1,25D downregulating GS and reducing glutamine utilization and dependence [104]. Additionally, UDP-glucose ceramide glucosyl transferase (UGCG) overexpression in breast cancer cells is associated with increased glutamine uptake and utilization, linking glycosphingolipid metabolism to glutamine metabolism [105]. Lastly, the EPHA2/ephrin-A1 signaling axis in regulating glutamine metabolism in HER2-positive breast cancer has been explored, with findings suggesting that ephrin-A1 loss leads to upregulated glutamine metabolism and lipid accumulation, enhancing tumor growth [106]. This is consistent with the broader understanding that glutamine is crucial for cancer cell proliferation and survival, with its role being influenced by the expression levels of oncogenes and tumor suppressors [107]. In the context of HER2-positive breast cancer, the receptor tyrosine kinase EphA2 has been found to promote glutamine metabolism by activating the transcriptional coactivators YAP and TAZ, suggesting potential therapeutic targets in this cancer subtype [108]. In our research, we found that exposing MCF-7 breast cancer cells to a glucose-deprived environment with abundant β-hydroxybutyrate activates the HIPPO pathway. This suggests that a ketogenic diet, which is currently being investigated as a therapeutic approach, may shift breast cancer cells toward compensatory glutamine catabolism [109].

### 2.6. Cell Signaling Pathway Dysregulation Affecting Glucose Metabolism

Glucose cannot enter the cell on its own due to the selective permeability of the cell membrane. In epithelial cells, glucose is transported into the cell through various glucose transporters. Among these, GLUT4 is unique because its presence on the cell surface is regulated by insulin signaling [110]. Additional glucose transporters are involved in glucose transport in epithelial cells, which are likely involved in carcinomas as well [111]. Thus, the regulation of glucose transporters and their related cell signaling pathways are involved in promoting alterations in breast cancer cell metabolism. The PI3K/AKT/mTOR pathway is a crucial signaling pathway that intersects with insulin signaling to regulate glucose uptake in epithelial cells. This pathway is particularly important in understanding the metabolic dysregulation seen in cancer and other diseases. In 3T3-L1 adipocytes, insulin activates the PI3K pathway, leading to the translocation of the glucose transporter GLUT4 to the plasma membrane, thereby increasing glucose uptake [112]. The PI3K/Akt/mTOR pathway plays a crucial role in breast cancer biology and pathogenesis, with its dysregulation contributing to tumor initiation, progression, and resistance to therapy [113,114,115]. In breast cancer, mutations in the PI3K pathway are common and contribute to tumor progression and resistance to therapies [116,117]. Aberrant activation of this pathway is linked to resistance to endocrine therapies and HER2-targeted treatments in breast cancer. Targeting the PI3K/AKT/mTOR pathway can help overcome this resistance and improve therapeutic outcomes [118,119]. The PI3K/AKT/mTOR pathway also plays a role in cell motility, migration, and invasion, contributing to the metastatic potential of breast cancer cells. This enhances the ability of cancer cells to spread and establish secondary tumors [120]. Inhibitors targeting the PI3K/AKT/mTOR pathway are under clinical investigation and have shown promise in treating breast cancer. Drugs like everolimus (an mTOR inhibitor) have been approved for use in certain breast cancers, highlighting the clinical relevance of targeting this pathway [113]. Despite initial challenges, the development of more precise PI3K inhibitors has shown clinical benefit, particularly in patients with advanced breast cancer [121]. Novartis Oncology is exploring various strategies to maximize the benefits of clinical studies with these inhibitors, including patient stratification and enrollment based on PI3K pathway activation status [122].

MYC is a crucial oncoprotein that significantly impacts the altered signaling pathways in cancer cells, including breast cancer. The overexpression of MYC plays a key role in the dysregulated metabolism of these cells. For instance, MYC overexpression increases the transcription of glycolytic enzymes and glucose transporters, such as GLUT1, phosphofructokinase, and enolase, which leads to enhanced glycolysis and lactate production [123]. MYC is also necessary for the glucose-mediated activation of glucose-responsive genes, like liver-type pyruvate kinase, through its interaction with the carbohydrate response element-binding protein (ChREBP) [124]. Additionally, *c-Myc* expression is regulated by glucose levels, where low glucose concentrations result in increased *c-Myc* mRNA levels [125]. This indicates a feedback loop in which *c-Myc* not only regulates glucose metabolism but is also influenced by it. This regulatory mechanism highlights *c-Myc*’s pivotal role in activating the metabolic networks required for cell proliferation, emphasizing its importance in cancer development and progression [126].

Moreover, MYC enhances glutamine metabolism by upregulating genes such as GLS1, which converts glutamine to glutamate, thereby supporting the TCA cycle and providing precursors for nucleotide and amino acid biosynthesis [127,128,129]. This reprogramming of glutamine catabolism is vital for maintaining cellular viability and ensuring a steady supply of intermediates for the TCA cycle [129]. MYC activation is also crucial for adenovirus-induced upregulation of host cell glutamine utilization, which promotes optimal virus replication [130]. Furthermore, MYC overexpression contributes to mitochondrial biogenesis and enhances OXPHOS, enabling cancer cells to adapt to metabolic stress and nutrient deprivation. This adaptability is facilitated by activating various metabolic pathways, including the PI3K/AKT/mTOR axis, and promoting glutaminolysis to support ATP production [61,131,132,133]. In breast cancer, targeting both glycolytic and glutaminolytic pathways has demonstrated significant potential in reducing tumor growth, with studies showing that combining inhibitors of these pathways with chemotherapy can significantly inhibit tumor proliferation [134].

### 2.7. Metabolic Alterations Due to Hypoxia-Inducible Factor (HIF) Activation

HIF activation, particularly the stabilization of HIF-1α under hypoxic conditions, is a key mechanism contributing to the dysregulated metabolism of cancer cells, including breast cancer. The stabilization of HIF-1α leads to the expression of glycolytic enzymes and glucose transporters, such as GLUT1 and LDHA, enhancing glycolysis and glucose uptake—hallmarks of cancer cell metabolism [135]. Under hypoxic conditions, HIF-1α promotes a shift towards anaerobic glycolysis by increasing the transcription of genes involved in glucose transport and glycolysis, which supports cancer cell survival in low-oxygen environments.

In addition to its metabolic effects, HIF-1α stabilization is associated with increased chemoresistance in breast cancer cells. For example, the protein anterior gradient 2 (AGR2) has been shown to stabilize HIF-1α, thereby enhancing resistance to the chemotherapeutic agent doxorubicin by reducing its degradation and promoting the expression of multidrug resistance proteins [136]. HIF-1α, whether induced by hypoxia or mutations in the VHL gene, reprograms cancer cell metabolism by increasing glucose transport and conversion to pyruvate while simultaneously decreasing mitochondrial metabolism and mass, thus shifting energy production away from mitochondria and towards glycolysis [137,138]. This shift not only promotes tumor cell survival in hypoxic microenvironments but also plays a role in regulating tumor angiogenesis and enhancing tumor progression and aggressiveness [139].

Furthermore, HIF-1α is implicated in the acquisition of metastatic behavior, suggesting a potential link between tumor cell hypoxia and metastasis [140]. The regulation of key metabolic enzymes, such as pyruvate kinase, also appears to be under the control of HIF-1α, which includes the upregulation of PDK1. PDK1 inhibits the conversion of pyruvate to acetyl-CoA, further diverting glucose metabolism towards glycolysis [59,141,142]. Prolonged stabilization of HIF-1α, even under normoxic conditions, can lead to mitochondrial adaptations that support cancer cell stemness and tumorigenicity. This includes the accumulation of metabolites like succinate and fumarate, which further stabilize HIF-1α and promote a metabolic state conducive to tumor growth [143]. Thus, the activation of HIF-1α significantly influences the metabolic reprogramming of breast cancer cells by promoting glycolysis, enhancing survival under hypoxic conditions, supporting metastatic potential, and contributing to therapeutic resistance.

### 2.8. Alterations in Metabolite Levels

The accumulation of altered metabolites, such as oncometabolites like 2-hydroxyglutarate (2HG), plays a critical role in the dysregulated metabolism of cancer cells, including breast cancer. These oncometabolites contribute to the acidic TME, which affects gene expression and cell differentiation, ultimately promoting malignancy. The oncometabolite 2HG inhibits α-ketoglutarate-dependent dioxygenases, such as histone and DNA demethylases, leading to widespread epigenetic changes that facilitate cancer progression [144,145,146,147]. This inhibition results in altered methylation of histones and DNA, causing gene silencing and the repression of differentiation-related genes [144].

The accumulation of 2HG in tumors, often driven by mutations in the *IDH1* and *IDH2* genes, reduces α-ketoglutarate levels and induces genome-wide histone and DNA methylation changes [145]. This accumulation enhances gene silencing through the inhibition of specific histone demethylases, contributing to tumor progression [146]. Additionally, elevated levels of 2HG are associated with the activation of the MYC pathway in breast cancer. MYC overexpression leads to increased 2HG levels, which, in turn, causes global DNA hypermethylation. This subtype of breast cancer, characterized by high levels of 2HG, is linked to poor prognosis and is more frequently observed in African American patients [148].

The oncogene *ADHFE1* also plays a role in this process, as it induces metabolic reprogramming and cellular dedifferentiation by driving the accumulation of 2HG in breast tumors. Regulated by MYC, *ADHFE1* enhances tumor growth and promotes a stem cell-like phenotype, thereby increasing tumor aggressiveness [149,150]. Elevated 2HG levels in breast cancer cells have been associated with a stem cell-like transcriptional signature and increased expression of epithelial-mesenchymal transition (EMT)-associated genes [148]. This finding is consistent with the research of Grassian and colleagues, who demonstrated that the accumulation of 2HG, often resulting from *IDH1/2* mutations, can induce an EMT-like phenotype [151]. The EMT process, which is closely linked to the acquisition of stem cell properties, is a key factor in breast cancer progression [152,153].

### 2.9. Effects of Altered Metabolism on the Tumor Microenvironment

One significant aspect of this altered metabolism is the production of lactate and protons, leading to an acidic TME. This acidic environment facilitates invasion, metastasis, and immune evasion in cancer cells, including breast cancer. Cancer cells adapt to the acidic microenvironment by upregulating monocarboxylate transporters (MCTs) and carbonic anhydrase IX (CAIX), which help to export excess lactate and protons, maintaining intracellular pH balance [154]. This upregulation is crucial for the maintenance of the hyper-glycolytic acid-resistant phenotype of cancer, allowing for high glycolytic rates and pH regulation [155]. The formation of a transport metabolon between CAIX and MCTs further supports this role in tumor metabolism and proliferation [156]. Targeting these pH regulators may offer the potential for more effective anticancer strategies [157,158].

The production of lactate by cancer cells creates an acidic TME that suppresses anticancer immunity by inhibiting the proliferation and function of immune cells like T lymphocytes [159,160]. This immunosuppression is further exacerbated by lactate’s role in recruiting and inducing immunosuppressive cell types in the TME [161]. Lactate also regulates immune responses by causing extracellular acidification, acting as an energy source, and inhibiting the mTOR pathway in immune cells [162]. Acidic conditions in the TME have been shown to induce an anergic state in CD8+ T cells, impairing their cytolytic activity and cytokine secretion and ultimately diminishing the immune response against tumors [163,164,165]. This anergy is characterized by reduced expression of IL-2Rα and T-cell receptors, as well as diminished activation of STAT5 and extracellular signal-regulated kinase (ERK) after TCR activation [163]. The dysfunction of CD8+ T cells in acidic conditions is further characterized by impaired IL-2 responsiveness, perturbations to mTORC1 signaling and *c-Myc* levels, and altered nutrient uptake and processing [164]. However, this anergic state is reversible, as the administration of proton pump inhibitors can buffer tumor acidity and restore T-cell function [165]. Overall, the interplay between extracellular acidosis and immune cells, particularly CD8+ T cells, is a critical factor in the immune response against tumors [166].

Additionally, the acidic microenvironment created by aberrant tumor metabolism plays a crucial role in shaping the function of tumor-associated macrophages (TAMs), with lactate and protons being key factors in this process [167,168]. Lactate can reprogram TAMs to adopt an immunosuppressive phenotype and promote angiogenesis, thereby supporting tumor growth [169]. Similarly, protons, which contribute to the acidic TME, can modulate the metabolism of TAMs and influence local and systemic immunity [170]. These findings underscore the importance of understanding the metabolic changes in TAMs and the TME for the development of novel therapeutic approaches targeting immune cell metabolism. In addition to TAMS and CD8+ T cells, acidic TME plays a crucial role in the induction of regulatory T cells (Tregs), which in turn suppress the anti-tumor immune response and promote tumor survival [159,171,172,173]. Lactate is a key factor in this process, as it can promote the differentiation of Tregs from conventional CD4+ T cells in a pH-dependent manner [171,172]. The TME’s acidity also supports the metabolic flexibility of Tregs, allowing them to use lactate as an alternative energy source and maintain their suppressive function [159,173]. These findings highlight the potential of targeting acidic TME as a therapeutic strategy to reduce Treg-mediated immune suppression and enhance anti-tumor immune responses.

Acidic microenvironments in breast cancer cells have been found to induce the expression of interleukin-8 (IL-8) and matrix metalloproteinases (MMPs), leading to enhanced migration and invasion capabilities. This process is mediated by acid-sensing ion channel 1 (ASIC1) and the ROS-AKT-NF-κB pathway, which are activated under acidic conditions [174,175]. The interaction of breast cancer cells with monocytes further enriches the acidic microenvironment, promoting the secretion of IL-8 and MMPs [176]. IL-8 has been shown to play a significant role in breast cancer progression, with high expression levels in ER- and HER2+ breast cancers [177]. Lactate in the TME induces the expression of GPR81, a lactate receptor, through a Snail/STAT3 pathway, promoting tumor growth and metastasis [178]. In highly glycolytic gastric cancer, lactate/GPR81 signaling recruits regulatory T cells, contributing to immune resistance [179]. GPR81 also promotes a malignant phenotype in breast cancer by enhancing angiogenesis through the PI3K/Akt-CREB pathway [180]. The lactate/GPR81 signaling axis is involved in various aspects of cancer progression, including angiogenesis, immune escape, and the Warburg phenomenon [181].

## 3. Organoid Models in Cancer Research

Organoids are derived from patient-derived tissues (PDO) or xenografts (PDxO), induced pluripotent stem cells (iPSC), or adult stem cells (ASC), which are typically given a scaffold and the necessary biochemical factors to induce or maintain the desired differentiation [182]. These models have greatly eliminated many traditional 2D-model issues and have better gene amplification stability, cell-to-cell, and pericellular matrix communication [183,184]. They offer greater cellular heterogeneity, a more diverse microenvironment, a larger array of cell interactions, and a greater degree of spatial organization [31]. Additionally, human-based organoids eliminate interspecies differences, reducing potential issues that may arise with animal models. For example, human gastric pathologies such as *Helicobacter pylori* infection are better studied in gastric organoid models as opposed to mouse models because, unlike in humans, the infection in mice does not progress to ulceration and cancer [185]. Using organoids in these and other studies has the potential to increase the efficacy of clinical and translational research while also easing animal model ethical and logistical problems [186]. This more physiologically relevant model allows for greater insight into tissue repair, development, and regeneration.

**Figure 3:**
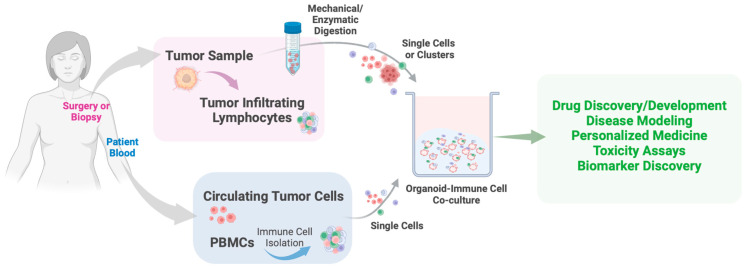
Schematic representation of PDTO-immune cell co-culture workflow and functional testing. PDTO-immune cell co-culturing begins with sourcing and digesting patient tumor samples to isolate clusters or single cancer cells and tumor-infiltrating lymphocytes (TILs) or by isolating circulating tumor cells and PBMCs from patient blood. The cells are then embedded in an ECM for structural support. Fully formed tumor organoids co-cultured with immune cells (macrophages, T cells, B cells, NK cells, etc.) may be used in drug discovery, personalized medicine, therapeutic toxicity studies, and biomarker discovery. Created with BioRender.com.

PDTOs are most commonly derived from tumor biopsies or circulating tumor cells [32] (Figure 3). Numerous PDTOs have been generated, such as prostate [187], pancreas [24,26], colon [188,189], and breast [190,191,192,193]. These organoids have been used in a wide array of studies, including those on metastasis [194], EMT [195], cell signaling [196], and metabolism [26,197,198].

In 2018, the Clevers group successfully cultured over 100 primary and metastatic breast cancer organoid lines covering all major gene-expression-based classifications and designed a robust protocol for their long-term culturing [199]. Since then, significant advances have been made in developing breast cancer PDTO subtypes, including luminal, HER2-positive, and TNBC [200,201]. For example, Dekkers and colleagues developed protocols for long-term culturing of all major breast cancer subtypes (TNBC, estrogen-positive/progesterone receptor (PR)-positive, and HER-2-positive) [202]. In 2024, Han and colleagues successfully established organoid lines from two surgical tumor specimens of a bilateral breast cancer (BBC) patient, likely originating from TNBC tissue [203]. They demonstrated that although these organoids represent diverse molecular subtypes, they retain significant markers and driver mutations from the original tumor.

PDTOs have been instrumental in identifying metabolic alterations in breast cancer. For example, Gong and colleagues used a multi-omics approach to systematically characterize metabolic reprogramming and the heterogeneity of TNBC tumor samples and PDTOs [204]. They identified three distinct metabolic-pathway-based subtypes of TNBC, each with unique metabolic characteristics and different prognoses, distributions of molecular subtypes, and genomic alterations. These subtypes include MPS1, a lipogenic subtype characterized by increased lipid metabolism; MPS2, a glycolytic subtype with elevated carbohydrate and nucleotide metabolism; and MPS3, a mixed subtype with partial dysregulation of various pathways. Similarly, Xiao and colleagues worked to characterize the metabolome of TNBC PDTOs, categorizing metabolomic subgroups, and providing a landscape of potential therapeutic targets using a transcriptomic approach [197]. These categories include C1, which describes cells by their enrichment of ceramides and fatty acids; C2, characterized by the upregulation of oxidation reaction and glycosyl metabolites; and C3, the category with the least metabolic dysregulation.

Recently, more advanced approaches, such as PDTO-immune co-culture models, have been developed to provide a more accurate representation of the in vivo TME [205,206] (Figure 3). Immune interactions play a crucial role in tumor metabolism and metabolic reprogramming, influencing how cancer cells adapt and survive under varying conditions. By capturing these dynamics, organoid-immune co-cultures offer valuable insights into how metabolic pathways can be targeted alongside immunotherapies. Numerous PDTO-immune co-culture models have been developed, including CRC [207], renal cell carcinoma [208], lung [209,210], and bladder [211], among others. Although breast tumor-immune cell co-culture models are still in their early stages, there have been promising attempts. For example, Aung and colleagues utilized a tumor-on-a-chip platform to create a microfluidic breast cancer-immune model [212]. This ex vivo system integrates various cell types, including cancer cells (MCF7), monocytes (THP-1), and endothelial cells. Briefly, T cells were introduced into perfused media and allowed to infiltrate the tumor model, with the presence of monocytes enhancing T cell recruitment through chemokine release. This microfluidics-based technique creates 3D tumor tissues with diverse microenvironmental features, offering valuable insights for clinical applications [212]. Recently, Raffo-Romero and colleagues developed three optimized methods for co-culturing human macrophages with breast cancer organoids: a semi-liquid model and two matrix-embedded models, in which they found that macrophages not only affected the organoid’s molecular profiles but also influenced chemotherapy responses [213]. And finally, Xu and colleagues developed a 3D co-culture of breast cancer cells and tumor-infiltrating macrophages [214]. Their co-culture model significantly improved breast cancer organoid growth and exhibited more aggressive cancer phenotypes, including enhanced stemness, migration, ECM remodeling, and cytokine secretion. Transcriptomic and protein–protein interaction analyses identified key pathways and targets involved in breast cancer progression within a macrophage-enriched immune environment.

## 4. Organoid-Based Approaches in Studying Breast Cancer Metabolism

Changes in cellular metabolism play a vital role in the progression of breast cancer, whereby tumors can utilize many different substrates (glucose, amino, or fatty acids) depending on their TME. Interpatient and intratumoral heterogeneity, characterized by diverse cellular compositions between patients and within individual tumors, leads to significant variability in metabolic processing and consequent disparities in treatment outcomes. Understanding the metabolic adaptations in breast cancer can help identify therapeutic targets. Use of PDTOs to investigate metabolic preferences in various tumor types with, often intact, original TME. Multiple methods have been applied in measuring metabolic activity in tumor organoids.

### 4.1. Optical Metabolic Imaging (OMI)

**Figure 4:**
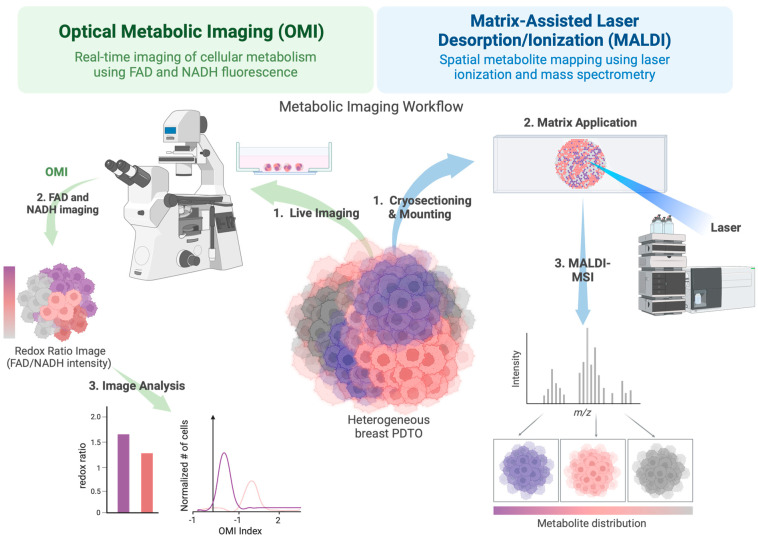
Metabolic imaging of breast cancer PDTOs using OMI and MALDI-MSI. Schematic representation of imaging workflow for assessing metabolic activity in breast cancer PDTOs using OMI and MALDI-MSI. In the OMI workflow (left), autofluorescence of NADH and FAD is used to monitor cellular metabolism. This involves the acquisition of live images (1) and generating a redox ratio image (2) that reflects the metabolic state of the cells. Finally, image analysis is performed to compute the redox ratio and OMI index, quantifying metabolic heterogeneity across the PDTO. In the MALDI-MSI workflow (right), PDTOs are prepared for mass spectrometry analysis. First, organoids are cryosectioned and mounted (1), followed by matrix application (2) to facilitate laser-based ionization. MALDI-mass spectrometry imaging is conducted (3), producing an *m*/*z* spectrum and spatially resolved metabolic heatmaps, illustrating metabolite distribution within the PDO. The integration of OMI and MALDI-MSI enables high-resolution metabolic profiling of PDOs, providing insights into tumor metabolic heterogeneity and potential therapeutic targets. Created with BioRender.com.

OMI is a powerful two-photon fluorescence lifetime imaging microscopy (FLIM) technique that exploits the auto-fluorescent properties of NAD(P)H and flavin coenzymes—flavin adenine dinucleotide (FAD) and flavin mononucleotide (FMN) to monitor cellular metabolism in more physiologically relevant conditions (Figure 4) [215]. The technique is highly sensitive, high-resolution, non-invasive, and quantitative. Using OMI, researchers measure (1) the optical redox ratio, i.e., the relative amount of electron donor and acceptor in the cell—a useful measure for how oxidized or reduced the cell itself is, and (2) the autofluorescence lifetime of NADH and FAD by measuring the amount of NADH and FAD in a free or protein-bound state due to changes in conformation upon binding. A key advantage of OMI is that NAD(P)H and FAD/FMN are involved in over 200 signaling pathways, including glycolysis, the TCA cycle, OXPHOS, fatty acid synthesis, glutaminolysis, and FAO. By leveraging the specific characteristics of NAD(P)H, FAD, and FMN auto fluorescent lifetimes and intensities, as well as responses to metabolic modulators, it is possible to distinguish between specific metabolic pathways.

The Skala group has pioneered the development of the OMI technique to detect distinct metabolic states within a range of tumor organoids, including colorectal [29], neuroendocrine [216], pancreatic [217], head and neck [218], and breast, and has also utilized OMI to predict therapeutic responses and assess drug efficacy in these models [30,217,219]. In a seminal study, Walsh and colleagues used OMI to detect unique metabolic changes in human breast cancer-derived xenografts (PDX) and PDxO within 24 h of a panel of anticancer drug treatment [219]. Briefly, in responsive cells, drug treatment leads to an immediate reduction in glycolysis rates and protein-binding of NADH and FAD, resulting in lower redox ratios followed by significant reductions in tumor growth 7–11 days post-treatment. These results were validated with in vivo tumor growth measurements, and resolved heterogeneous cellular responses, highlighting OMI’s potential as a high-throughput tool for investigating drug responses. Building upon this work, Sharick and colleagues utilized single-cell tracking by OMI to capture the metabolic heterogeneity within breast cancer organoids, demonstrating distinct metabolic profiles that corresponded with different drug responses, which may provide a novel approach to predict the presence of potentially treatment-resistant tumor cell populations within a largely responsive tumor cell population, which might otherwise be overlooked by traditional measurements [198]. The detection of metabolic subpopulations of drug-resistant cells within organoids, predicting long-term tumor drug response, has been confirmed in breast cell line xenografts [219,220]. OMI has also recently been used to analyze macrophage metabolic heterogeneity within 3D breast TME models. A study by Heaster and colleagues successfully captured spatiotemporal changes in macrophage metabolism, polarization, and migration in both 2D and 3D cultures, including 3D microfluidic co-cultures with breast cancer cells, revealing significant metabolic differences and enhanced migration compared to monocultures [221]. By providing single-cell resolution insights into macrophage dynamics in the TME, this method advances our understanding of tumor-immune interactions and macrophage behavior in cancer research.

Although powerful, OMI has several limitations. The penetration depth and opacity of multiphoton imaging of tightly organized cell layers can make it challenging to image the entirety of larger or more densely packed organoids. Moreover, prolonged exposure to excitation light can cause photobleaching of the fluorescent signals and phototoxic effects on the cells, potentially altering the metabolic state of the organoids during imaging.

### 4.2. Matrix-Assisted Laser Desorption/Ionization Mass Spectrometry Imaging (MALDI-MSI)

MALDI-MSI is a mass spectrometry-based technique for visualizing the spatial distribution of metabolites within biological samples and has been increasingly applied to study the metabolic heterogeneity of cancer, including the use of tumor organoids (Figure 4). While traditional MS analyzes a sample, providing an overall spectrum of detected molecules, MALDI-MSI uses a laser to sequentially scan the sample’s surface, ionizing molecules at specific locations. This allows for the generation of detailed images that show where specific molecules are found within the sample, providing spatial context that is absent in conventional MS. This approach offers the unique advantage of mapping the metabolic landscape in situ, preserving the spatial context crucial for understanding tumor heterogeneity. As a spatial metabolomic technique, MALDI-MSI has been used to classify cancer tissue subtypes using altered metabolites information and identify key metabolites in breast cancer studies [222]. In 3D tissue applications, the Hummon group developed a method to utilize MALDI-MSI to investigate protein distributions in a colon carcinoma spheroid model [223]. MALDI-MSI has since been used extensively to investigate protein, lipid, phospholipid, and metabolite distribution, chemotherapeutic drug penetration, liposome drug delivery, and proteomic changes in colon carcinoma spheroids [224,225,226,227,228,229,230], as well as skin [231,232], blood-brain-barrier [233], pancreatic [234], and breast cancer organoids [235].

Illustrating its usefulness in studying breast cancer metabolic dysregulation, a study by the Giampà group used MALDI-MSI to investigate lipid alterations in patient-derived breast cancer xenograft FFPE tissue. Briefly, the study investigated the spatial metabolic differentiation within specific tissue compartments and treatment responses induced by CB-839, a glutaminase inhibitor that has demonstrated antiproliferative activity in TNBC [236,237]. The group identified specific metabolic lipid alterations, altered levels of specific lipids, and reduced heterogeneity between xenograft breast tumors treated with CB-839 compared to the control tumors. Torata and colleagues used MALDI-MSI to characterize breast carcinoma tissues embedded in frozen tissue microarrays by analyzing energy charge and adenosine phosphate compound values [238]. They found that breast carcinoma tissues have higher energy charge and adenosine phosphate compound values than normal and concluded that MALDI-MSI could be a useful tool for analyzing breast carcinoma. Using breast cancer spheroids as a model, Tucker and colleagues utilized MALDI-MSI and high spectral resolution Fourier-transform ion cyclotron resonance (FT-ICR) to image endogenous metabolite distribution [239]. Their work showed that the spatial localization of adenosine phosphate and glutathione in the central region of breast cancer spheroids could serve as markers for increased hypoxic and oxidative stress.

MALDI-MSI is still in its early stages of application in breast tumor organoid studies, particularly in exploring metabolic reprogramming. Its potential usefulness is significant due to its ability to provide spatially resolved metabolic data, which is crucial for understanding the heterogeneity of metabolic alterations within tumors. However, MALDI-MSI has multiple limitations, including limited sensitivity to low-abundance species, complex data interpretation, fine structural resolution, cost, and accessibility.

### 4.3. High-Throughput Analytic Chemistry-Based Metabolic and Lipidomic Profiling

Nuclear magnetic resonance (NMR), liquid chromatography- (LC), and gas chromatography (GC)-mass spectrometry (MS) are powerful high-throughput analytical techniques that have recently been applied alone or in combination with other techniques to study metabolic reprogramming and measure tumor organoid metabolites [240,241].

For example, liquid chromatography-quadrupole time-of-flight mass spectrometry (LC-qTOF-MS), a highly sensitive analytical technique that combines separation by LC with high-resolution qTOF-MS, allows researchers to identify and quantify a vast array of metabolites and lipids with high accuracy due to its two distinct scan types for data acquisition—quadropole technology and a time-of-flight analyzer. In breast cancer research, Lackner and colleagues found that PI3K inhibition by specific PI3Kβ inhibitor AZD8186 in MDA-MB-468 breast cancer cells leads to significant changes in central metabolism pathways, as well as hexosamine and pyrimidine metabolism, revealing key metabolic processes associated with cancer metabolism and therapy [242]. Recently, protocols for utilizing LC-qTOF-MS to analyze drug-induced changes in tumor organoid metabolic profiles have been developed. The Haag group established a novel protocol for metabolomic and lipidomic profiling of colorectal cancer (CRC) organoids by LC-QTOF-MS that captures metabolic information from a minimal sample amount embedded in an ECM. As a proof of concept, they investigated the metabolic response of CRC organoids to 5-fluorouracil treatment and discovered expected dose-dependent changes in the metabolic profiles of metabolites involved in purine and pyrimidine metabolism [243]. Since then, this technique has been used to investigate metabolite changes in metastatic renal carcinoma [244], CRC [245], peritoneal tumor organoids [246], and others.

### 4.4. Other Technologies

Although advanced technologies for studying metabolic alterations are available, their application in three-dimensional cultures remains poorly optimized. As previously mentioned, oxygen concentration plays a critical role in mediating various cellular processes, such as metabolism, differentiation, and cell signaling. While multiple methods for studying oxygen consumption exist, they are still in the process of being optimized for use in 3D cultures.

The Agilent Seahorse Analyzers measure oxygen consumption rate (OCR) and proton efflux rate (PER)/extracellular acidification rate (ECAR) in real-time, making it a powerful tool for quantifying changes in OXPHOS and glycolysis. Recently, the Vanoni group optimized Seahorse metabolic analysis for high-resolution metabolic characterization of MCF7 and MDA-MB-231 breast tumor spheroid models [247]. Grün and colleagues developed novel microcavity arrays that allow the determination of oxygen in the microenvironment of organoids [248]. Nashimoto and colleagues developed a method where scanning electrochemical microscopy can non-invasively identify colorectal PDO subpopulations with different growth capabilities based on oxygen metabolism [249]. Building upon this work, the Shiku group used scanning electrochemical microscopy to determine OCRs of breast cancer cells in hydrogel fibers fabricated using an extrusion 3D bioprinter [250]. Dornhof and colleagues created a microfluidic platform for 3D cultivation of breast cancer spheroids, incorporating embedded electrochemical sensors for highly stable, long-term, real-time measurement of metabolic parameters, such as oxygen and lactate [251].

These advances highlight the ongoing efforts to enhance the applicability of metabolic studies in 3D cultures (Table 1). As these methods continue to evolve, they will provide deeper insights into the metabolic dynamics within complex tissue models. Ultimately, this progress will contribute to more effective strategies for studying disease and developing targeted therapies against metabolic vulnerabilities within breast cancer.

## 5. Conclusions

Organoids are important tools in cancer research for drug and immunotherapy discovery, screening, and validation, as well as biomarker identification and disease mechanism studies. Using tumor organoids has enhanced our understanding of metabolic reprogramming, offering a more physiologically relevant model compared to traditional two-dimensional cell cultures. These models allow for a closer resemblance to the TME in vivo, capturing the complex metabolic interactions and heterogeneity inherent in cancer. Recent advancements, such as the integration of various cell types (e.g., immune cells), further enhance their relevance for disease mechanism, personalized medicine, and high-throughput drug screening. Metabolic alterations are hallmarks of cancer development and present in most cancer types, giving the potential for widely applicable treatment opportunities. Techniques covered in this review, such as OMI, MALDI-MSI, and LC-qTOF-MS, provide powerful tools for investigating these metabolic processes, offering detailed insights into the spatial distribution of metabolites and the dynamic metabolic changes within tumor organoids. These technologies have not only improved our ability to study metabolic reprogramming in breast cancer but also hold promise for identifying novel therapeutic targets and predicting treatment responses with greater accuracy. For instance, by identifying specific metabolic pathways or subpopulations of cells that are resistant to current therapies, new drug targets can be identified, and treatment strategies can be developed. This understanding of tumor metabolism opens up possibilities for more precise and effective interventions, ultimately aiming to improve patient outcomes in breast cancer treatment.

However, it is important to acknowledge the limitations of 3D organoid models. A significant challenge is the lack of a standardized approach to studying and acquiring organoids, which stems from the absence of common protocols and highlights the need for biobanks that can widely distribute iPSC, ASC, or PDXs of the same disease derived from different patients [186]. The cultivation of PDTOs is prone to contamination and varying laboratory successes due to inconsistent methodologies. Although organoid heterogeneity offers potential benefits for personalized medicine, clinicians currently lack efficient tools to assess this variability [184]. Additionally, while current organoid models often lack vasculature, which is crucial for accurately simulating tumor metabolism and drug delivery, recent studies have shown promise in co-culturing human endothelial cells with breast tumor organoids to develop functional capillary networks [252]. This advancement, along with other experimental strategies to generate vascularized organoids, may further enhance the utility of organoids in metabolic studies. This gap complicates the discovery of novel drugs and the evaluation of cellular mechanisms, such as cell death or recurrence, within heterogeneous organoids. Despite these challenges, continued refinement of these models and methods will be crucial for advancing breast cancer research and developing more effective, tailored treatments.

## Figures and Tables

**Table 1:** Summary of recent organoid-based approaches in studying cancer metabolism.

Methodology	Organoid Model	Reference	Summary
Agilent Seahorse Analyzer	MCF7 and MDA-MB-231 breast tumor organoids	Campioni et al. (2022) [247]	Optimized Seahorse metabolic analysis for high-resolution metabolic characterization of breast cancer spheroids.
LC-qTOF-MS	Metastatic clear cell renal cell carcinoma (ccRCC) PDOs	Reustle et al. (2022) [244]	Studied metabolite changes in metastatic renal carcinoma organoids using LC-qTOF-MS.
LC-qTOF-MS	HCT116 and HT29 CRC organoids	Zhou et al. (2022) [245]	Investigated metabolite changes in CRC organoids, revealing insights into metabolic reprogramming.
LC-qTOF-MS	CRC PDOs—Ex vivo peritoneum co-cultures	Mönch et al. (2021) [246]	Analyzed metabolic profiles in CRC PDO-peritoneum co-cultures
LC-qTOF-MS	CRC PDOs	Neef et al. (2020) [243]	Developed a novel protocol for metabolomic and lipidomic profiling, identifying dose-dependent changes in metabolic profiles of CRC PDOs.
MALDI-MSI	Patient-derived breast cancer xenograft FFPE tissue	Denti et al. (2021) [237]	Investigated lipid alterations and treatment responses in breast cancer xenografts, identifying specific metabolic lipid changes and reduced heterogeneity with treatment.
MALDI-MSI	MCF7 breast tumor organoids	Tucker et al. (2019) [239]	Used MALDI-MSI to image endogenous metabolite distribution, identifying markers of hypoxic and oxidative stress in breast cancer spheroids.
MALDI-MSI	Breast carcinoma tissues embedded in frozen tissue microarrays	Torata et al. (2018) [238]	Analyzed energy charge and adenosine phosphate compound values in breast carcinoma tissues, finding higher values compared to normal tissue.
Microcavity arrays for oxygen concentration measurements	HCC spheroids	Grün et al. (2023) [248]	Developed microcavity arrays for determining oxygen in the organoid microenvironment.
Microfluidic platform with electrochemical sensors	TNBC PDTOs	Dornhof et al. (2021) [251]	Created a microfluidic platform for real-time measurement of metabolic parameters in breast cancer spheroids.
OMI	Primary invasive ductal carcinoma breast PDTO-macrophage co-cultures	Heaster et al. (2020) [221]	Captured spatiotemporal changes in macrophage metabolism, polarization, and migration in breast cancer organoid models, revealing significant metabolic differences.
OMI	Breast cancer PDxOs	Sharick et al. (2019) [198]	Demonstrated distinct metabolic profiles within breast cancer organoids, correlating with drug responses and identifying potentially treatment-resistant cell populations.
OMI	Breast cancer PDX and PDxOs	Walsh et al. (2014) [219]	Used OMI to detect metabolic changes in breast cancer organoids upon anticancer drug treatment.
Scanning electrochemical microscopy	3D breast cancer cell culture in hydrogel fibers	Kosuke et al. (2024) [250]	Determined oxygen consumption rates in breast cancer cells using scanning electrochemical microscopy in 3D bioprinted hydrogel fibers.
Scanning electrochemical microscopy	CRC PDOs	Nashimoto et al. (2023) [249]	Identified subpopulations with different growth capabilities based on oxygen metabolism in colorectal cancer organoids.

## Data Availability

Not applicable.
